# Draft Genome Sequences of Three *Lactobacillus* *paracasei* Strains, Members of the Nonstarter Microbiota of Mature Cheddar Cheese

**DOI:** 10.1128/genomeA.00655-17

**Published:** 2017-07-20

**Authors:** Ewelina Stefanovic, Gerald Fitzgerald, Olivia McAuliffe

**Affiliations:** a Teagasc Food Research Centre, Moorepark, Fermoy, Co. Cork, Ireland; b School of Microbiology, University College Cork, Cork, Ireland

## Abstract

*Lactobacillus paracasei* strains are common members of the nonstarter microbiota present in various types of cheeses. The draft genome sequences of three strains isolated from mature cheddar cheeses are reported here.

## GENOME ANNOUNCEMENT

Lactic acid bacteria (LAB) are Gram-positive aerotolerant bacteria with a wide spectrum of practical applications, including food production, biotechnology, and medicine-related fields (1). Strains of the genus *Lactobacillus* have been isolated from diverse habitats, such as fermented products, plant materials, and human and animal gastrointestinal tracts (2). In cheese, *Lactobacillus paracasei* forms part of the nonstarter microbiota and is considered to have an important role in the ripening process and flavor development (3). The three *Lactobacillus paracasei* strains (DPC2071, DPC4206, and DPC4536) analyzed in this study were isolated from mature cheddar cheeses as part of the nonstarter LAB population.

Bacterial DNA was isolated from all three strains, and genomic libraries were prepared with the Nextera XT DNA library preparation kit (Illumina, Inc., USA). The 2 × 250-bp paired-end read sequencing was performed on an Illumina MiSeq platform (MicrobesNG, University of Birmingham, United Kingdom). The assembly of each genome was performed using the SeqMan NGen application of the DNAStar Lasergene Genomics Suite (DNAStar, Inc., Madison, WI, USA). Glimmer version 3.02 (4) and RAST (5) were used to predict open reading frames (ORFs). Initially, the RAST server was used to annotate each genome, and the annotations were verified and manually curated using BLASTp (6) and Artemis (7).

Sequence assemblies for the three strains indicated coverages of 88×, 70×, and 101× for DPC2071, DPC4206, and DPC4536, respectively. The length of the DPC2071 genome was 2,936,872 bp, consisting of 41 nonoverlapping contigs, with a contig *N*_50_ of 300,051 bp, a maximum contig size of 536,232 bp, and a total of 2,827 protein-coding genes. In the case of strain DPC4206, the assembly yielded a genome sequence of 3,095,268 bp, consisting of 49 contigs. The maximum contig size was 322,047 bp, and the contig *N*_50_ was 142,300 bp, while a total of 2,951 protein-coding genes were identified. The draft genome sequence of strain DPC4536 was 3,078,575 bp long, and it consisted of 35 contigs and 2,931 genes encoding proteins. The maximum contig size was 426,277 bp, and the contig *N*_50_ was 191,696 bp. The G+C content of all three genomes was 46.3%, which corresponds to the usual G+C content of *L. paracasei* genomes.

These sequencing data will contribute to the pool of available *Lactobacillus paracasei* genomes and enable further comparative genome analysis of strains of this species.

### Accession number(s).

The whole-genome shotgun projects have been deposited at DDBJ/EMBL/GenBank under the accession numbers NCSN00000000, NCSO00000000, and NCSP00000000, while the versions described in this paper are versions NCSN01000000, NCSO01000000, and NCSP01000000 for strains DPC2071, DPC4206, and DPC4536, respectively.
